# Long-Term Impact of Guselkumab on Systemic Inflammation Indices in Moderate-to-Severe Psoriasis

**DOI:** 10.3390/jcm15020439

**Published:** 2026-01-06

**Authors:** Edoardo Mortato, Lorenzo Marcelli, Agostino Panichelli, Marina Talamonti, Valerio Gneo, Domenico Marrapodi, Cosimo Di Raimondo, Luca Bianchi, Marco Galluzzo

**Affiliations:** 1 Department of Systems Medicine, University of Rome “Tor Vergata”, 00133 Rome, Italy; edoardo.mortato@gmail.com (E.M.); lmarcelli96@gmail.com (L.M.); valerio.gn98@gmail.com (V.G.); domenicomarrapodi93@gmail.com (D.M.); luca.bianchi@uniroma2.it (L.B.); 2 School of Medicine and Surgery, University of Rome “Tor Vergata”, 00133 Rome, Italy; agostinopanichelli@gmail.com; 3 Dermatology Unit, Fondazione Policlinico “Tor Vergata”, 00133 Rome, Italy; talamonti.marina@gmail.com (M.T.); cosimodiraimondo@gmail.com (C.D.R.)

**Keywords:** psoriasis, guselkumab, biologic therapies, anti-IL-23, inflammatory indices

## Abstract

**Background/Objectives**: Psoriasis is a chronic immune-mediated inflammatory disease associated with systemic inflammation and comorbidities such as cardiovascular disease and metabolic syndrome. Blood-derived inflammatory indices like neutrophil-to-lymphocyte ratio (NLR), platelet-to-lymphocyte ratio (PLR), systemic immune-inflammation index (SII), systemic inflammation response index (SIRI), and pan-immune-inflammation value (PIV) have been proposed as biomarkers of systemic inflammation and disease severity. This retrospective and prospective observational study aimed to evaluate the long-term effects of guselkumab, an IL-23 inhibitor, on these indices in moderate-to-severe psoriasis. **Methods**: We analyzed 208 patients with moderate-to-severe psoriasis treated with guselkumab, with hematologic evaluations available for 208 patients at baseline, 208 at week 52, 129 at week 104, and 94 at week 156. Systemic inflammatory indices were calculated from routine annual blood tests. Patients were stratified by obesity, cardiovascular comorbidities, treatment response, and prior biologic therapy. Longitudinal changes were assessed using Friedman tests with Wilcoxon post hoc comparisons, and correlations between PASI and inflammatory indices were evaluated using Spearman’s coefficients. **Results**: SIRI and PLR showed significant reductions at week 156 (*p* = 0.038 and *p* = 0.018, respectively), while MLR also decreased over time without reaching consistent significance. NLR and PIV showed minimal or inconsistent changes. Obese patients and those with cardiovascular disease had higher baseline SII and SIRI and less pronounced improvements. No significant differences were observed between super responders and others. Correlation between baseline PASI and most inflammatory markers was weak, except for a weak but significant correlation with PIV (ρ = 0.119, *p* = 0.049). **Conclusions**: Guselkumab treatment is associated with long-term reduction in systemic inflammatory indices, particularly SIRI. The weak correlation of these markers with skin severity highlights a dissociation between cutaneous and systemic inflammation. SIRI and SII may serve as useful biomarkers to monitor systemic inflammation and guide comprehensive management in psoriasis patients.

## 1. Introduction

Psoriasis is a chronic, immune-mediated inflammatory disease affecting both the skin and systemic health [1,2]. Elevated levels of proinflammatory cytokines, including tumor necrosis factor (TNF)-α, interleukin (IL)-17, and IL-23, play a central role in disease pathogenesis and systemic involvement [3,4,5]. Systemic inflammation in psoriasis has been associated with a higher risk of comorbidities, such as cardiovascular disease and metabolic syndrome [6,7,8], highlighting the need for reliable biomarkers that reflect disease activity and treatment response [9,10,11]. Among proposed biomarkers, inflammatory indices derived from routine blood tests—such as neutrophil-to-lymphocyte ratio (NLR), platelet-to-lymphocyte ratio (PLR), systemic immune-inflammation index (SII), and systemic inflammation response index (SIRI)—have shown promise in detecting systemic inflammation and correlating with disease severity [12,13]. However, these indices present important limitations, including low disease specificity, susceptibility to confounding factors (e.g., infections, metabolic conditions, medications), and the absence of standardized cutoff values, which may restrict their routine clinical applicability [13]. Elevated SII and SIRI have been linked to obesity, metabolic comorbidities, and severe disease, while higher baseline SII may predict the need for biologic therapy switching, suggesting potential prognostic value [14,15,16,17]. Biologic therapies targeting TNF-α, IL-17, and IL-23 have been shown to reduce these inflammatory indices within the first months of treatment [17]. However, long-term effects, particularly of guselkumab—a selective IL-23 inhibitor with proven long-term efficacy and a favorable safety profile—remain poorly characterized [18,19]. This study aimed to evaluate the long-term effect of guselkumab on systemic inflammatory indices in patients with moderate-to-severe psoriasis and to explore their potential role as biomarkers in different clinical subgroups.

## 2. Materials and Methods

### 2.1. Study Design and Setting

We performed a retrospective and prospective observational analysis at the Dermatology Unit, Policlinico Tor Vergata, Rome, aimed at evaluating the long-term effect of guselkumab on systemic inflammatory indices in patients with moderate-to-severe plaque psoriasis. The study was conducted in a real-world clinical practice setting and included patients treated between 2019 and 2025.

### 2.2. Patient Population and Inclusion/Exclusion Criteria

Adult patients (≥18 years) treated with guselkumab for at least 3 years at the approved dosage of 100 mg subcutaneously at week 0, week 4, and every 8 weeks thereafter were included. The study was conducted in a real-world cohort, with data collection closed in June 2025. Patients were required to have at least 3 years of clinical follow-up. Patients were eligible if hematological data required for calculating inflammatory indices were available at week 52 and at least one additional predefined annual timepoint (week 104 or week 156). Patients with less than 3 years of follow-up, or without hematological data at week 52 and at least one subsequent timepoint, were excluded from the analysis. Demographic (age, sex, BMI), clinical (disease duration, comorbidities, previous biologic therapy, treatment response), and laboratory data (complete blood counts) were collected at baseline and annually thereafter until week 156.

### 2.3. Inflammatory Indices and Laboratory Measures

Systemic inflammatory indices were calculated at each timepoint, including NLR, PLR, MLR, SII, SIRI, and PIV. NLR reflects systemic inflammation and immune balance, PLR reflects inflammatory and thrombotic status, MLR indicates chronic inflammation and immune activation, SII combines neutrophil, lymphocyte, and platelet counts to quantify systemic inflammatory burden, SIRI integrates neutrophil, monocyte, and lymphocyte counts, and PIV is a composite marker including neutrophils, lymphocytes, monocytes, and platelets [20,21,22]. These indices were used as surrogate markers of systemic inflammation; classical inflammatory biomarkers (e.g., CRP, IL-6, TNF-α) were unavailable, limiting mechanistic interpretation. Formulas for all indices are reported in Supplementary Table S1.

### 2.4. Study Outcomes

The primary aim was to assess longitudinal changes in systemic inflammatory indices over time, while secondary analyses explored whether clinical characteristics—such as treatment response (super responders achieving PASI100 within 20 weeks vs. non-super responders), obesity (BMI ≥ 30 vs. <30), cardiovascular comorbidities (presence vs. absence), and prior biologic exposure (bio-experienced vs. biologic-naïve)—influenced inflammatory trajectories.

### 2.5. Statistical Analysis

Descriptive statistics were used to summarize population characteristics, with medians and interquartile ranges reported for continuous variables. Distribution normality was assessed using the Shapiro–Wilk test. Longitudinal comparisons were performed using the Friedman test for repeated measures, with Wilcoxon signed-rank tests for pairwise comparisons versus baseline, both for the overall population and for each predefined subgroup. Spearman’s rank correlation coefficient evaluated relationships between baseline PASI and systemic inflammatory indices. A *p*-value ≤ 0.05 was considered statistically significant. No correction for multiple testing was applied; therefore, borderline results should be interpreted cautiously. Sample size estimation for longitudinal analysis was conducted using G*Power software (3.1.9.6), assuming a medium effect size, four repeated measures, alpha = 0.05, and 80% power, indicating a minimum of 36 evaluable patients.

### 2.6. Ethics Approval

Ethical approval was obtained from the “COMITATO ETICO INDIPENDENTE FONDAZIONE POLICLINICO TOR VERGATA” (Registro Sperimentazioni 46.23—Parere 31 March 2023). The study adhered to established guidelines, and all patients provided written informed consent for retrospective use of clinical and demographic data, in accordance with the Declaration of Helsinki (1964 and later amendments).

## 3. Results

### 3.1. Systemic Inflammatory Indices in the Overall Population

In the overall population, 208 patients were included at baseline. Available data were present for 208 patients at week 52, 129 patients at week 104, and 94 patients at week 156. The cohort was predominantly male (60.8%), with a mean age of 55 years and a mean BMI of 27.8. Comorbidities included obesity in 29.4% of patients (BMI ≥ 30), hypertension in 35.5%, dyslipidemia in 37.1%, history of cardiac disease in 8.1%, and diabetes in 14.4%. Additionally, 65.3% of patients had received at least one prior biologic therapy. It should be noted that long-term results at week 156 are based on less than half of the initial cohort, introducing a potential selection bias that is considered in the discussion. Longitudinal analysis of systemic inflammatory indices revealed heterogeneous patterns. NLR did not change significantly over time (Friedman *p* = 0.335), with median values remaining stable. PLR showed a significant overall reduction (*p* = 0.018), with a notable decline at week 156 compared to baseline (median from 115 to 108). MLR decreased significantly at week 156 (*p* = 0.009), although the global trend was not statistically significant. PIV showed a significant decrease at week 52 (*p* = 0.032), but no sustained change thereafter (overall *p* = 0.531). SII demonstrated a borderline trend toward reduction (*p* = 0.055) without significant changes at individual time points. SIRI showed a trend towards reduction over time (*p* = 0.078), which did not reach conventional statistical significance, and should therefore be interpreted cautiously. Complete results are presented in Table 1. In all tables, data are presented as medians with interquartile ranges (25th–75th percentiles), and statistically significant results are highlighted in bold.

### 3.2. Obesity Subgroup (BMI ≥ 30 vs. Normal Weight)

A total of 77 obese patients were included at baseline and week 52, 41 at week 104, and 34 at week 156; the remaining patients in each timepoint represented the non-obese group (131, 88, and 60 patients, respectively). Obese patients had higher baseline SII and SIRI compared to non-obese individuals, reflecting a higher systemic inflammatory burden. Longitudinal trends were generally similar between groups; statistically significant reductions in PLR (*p* = 0.039) and SII (*p* = 0.014) were observed only in non-obese patients, as expected, highlighting that obesity represents a confounding factor that may blunt or delay the systemic anti-inflammatory response to guselkumab (Table 2).

### 3.3. Cardiovascular Comorbidity Subgroup

Patients with cardiovascular comorbidities included 24 subjects at baseline and week 52, 9 at week 104, and 8 at week 156; the corresponding non-cardiopathic groups comprised 184, 120, and 86 patients, respectively. Patients with cardiovascular comorbidities had elevated baseline PIV and SII values, and SIRI remained persistently high throughout the study. Despite this, a general decline in inflammatory markers over time was observed regardless of comorbidity status (Table 3).

### 3.4. Treatment Response Subgroup (Super Responders vs. Non-Super Responders)

Super Responders (SR) were defined as patients achieving PASI100 within 20 weeks; all remaining patients were classified as Non-Super Responders (nSR). Thus, nSR did not include “non-responders only”, but all patients who did not achieve PASI100 within the predefined timeframe. The SR population consisted of 126 patients at baseline and week 52, 69 at week 104, and 48 at week 156; the nSR group included 82, 60, and 46 patients at the same timepoints. No significant differences in systemic inflammatory indices were observed between Super Responders (SR) and Non-Super Responders (nSR). Both groups followed similar trajectories, suggesting that complete skin clearance does not necessarily correlate with systemic inflammation control (Table 4).

### 3.5. Biologic Experience Subgroup (Biologic-Naïve vs. Biologic-Experienced)

The biologic-experienced group included 136 patients at baseline and week 52, 80 at week 104, and 56 at week 156; the biologic-naïve group comprised 72, 49, and 38 patients, respectively. Both biologic-naïve and biologic-experienced patients exhibited a consistent decline across all indices. MLR demonstrated a statistically significant reduction in both groups (*p* = 0.045 and *p* = 0.036, respectively), supporting a general trend toward improved systemic inflammation control under guselkumab treatment (Table 5).

### 3.6. Correlation Between PASI and Systemic Inflammatory Markers

Correlation analysis at baseline revealed generally weak associations between PASI and systemic inflammatory markers (Table 6). Spearman’s correlation coefficients were low and non-significant for most markers: NLR (ρ = 0.058, *p* = 0.358), PLR (ρ = 0.082, *p* = 0.195), MLR (ρ = 0.106, *p* = 0.092), SII (ρ = 0.075, *p* = 0.233), SIRI (ρ = 0.121, *p* = 0.100). Only PIV showed a weak but statistically significant correlation with PASI at baseline (ρ = 0.119, *p* = 0.049). These findings highlight a potential dissociation between skin severity and systemic inflammation in psoriasis.

## 4. Discussion

In this real-world study, we investigated the long-term trajectory of systemic inflammatory indices in patients with moderate-to-severe plaque psoriasis treated with guselkumab over a period of up to 156 weeks, observing an overall trend toward reduction in several markers—particularly PLR, MLR, and SIRI—supporting the hypothesis that IL-23 inhibition exerts sustained systemic anti-inflammatory effects. However, the response across indices was heterogeneous, with NLR and PIV showing minimal or inconsistent variations over time, while SIRI emerged as the most reliable marker of long-term inflammation control. These findings align with the growing body of literature supporting the relevance of hematologic systemic inflammatory indices as accessible biomarkers in psoriasis. Zhao et al. demonstrated a significant association between SII and psoriasis in a large NHANES cohort, particularly among obese patients and women, showing a dose–response relationship [14]. Similarly, Yang et al. confirmed that elevated SII was linked to increased psoriasis risk across multiple subgroups and displayed non-linear relationships with disease susceptibility [12]. Evidence from clinical cohorts further reinforces the elevated inflammatory profile in psoriatic patients, as shown by Solak et al., who found significantly higher SII, SIRI, NLR, PLR, and other ratios in psoriasis compared to healthy controls, with more marked elevations in severe disease [15]. Such observations are consistent with our baseline findings, where obese patients and those with cardiometabolic comorbidities presented higher systemic inflammatory indices. In particular, SIRI appears to be the index most strongly associated with both cardiovascular and metabolic comorbidities [23,24]. Tamer et al. showed that multiple biologics—including adalimumab, infliximab, ixekizumab, secukinumab, ustekinumab, and risankizumab—significantly reduced SII and SIRI after three months of therapy [17]. In that study, SIRI was higher at baseline in males, obese patients and individuals with more severe disease, and tended to be increased in patients with cardiometabolic comorbidities; importantly, it showed a consistent decline after biologic treatment across different drug classes, further supporting its role as a sensitive marker of systemic inflammatory burden and treatment response. Moreover, risankizumab, another IL-23p19 inhibitor, has demonstrated progressive reductions in CRP, ESR, NLR, SII, and SIRI over 52 weeks in a prospective multicentre observational study, with the most pronounced anti-inflammatory effects observed in very early responders. In the same cohort, further hematological modulation was also observed—including decreases in neutrophils and mean platelet volume, transient eosinophil increases at week 12, and a progressive basophil elevation—supporting the concept that IL-23 blockade induces dynamic, time-dependent changes in peripheral inflammatory profiles [25].

Importantly, Phase III trials have directly assessed the effect of guselkumab on systemic inflammation. In post hoc analyses of VOYAGE I, VOYAGE II, and ECLIPSE, involving more than 2800 randomized patients, guselkumab significantly decreased NLR, PLR, and MLR at Week 16 compared to placebo (VOYAGE I and II), with similar reductions to secukinumab and smaller reductions than adalimumab [19]. These biomarkers showed weak correlations with PASI and modest correlations with CRP, indicating that systemic inflammation may improve independently of cutaneous response. Our study extends these data by showing that SIRI reduction is sustained over three years in real-world settings, whereas other markers show less durable trends. Consistent with the findings of Tamer et al. [17], obese and cardiovascular patients in our cohort exhibited higher baseline SIRI values and a less pronounced decline over time, suggesting that SIRI may capture persistent low-grade systemic inflammation that is only partially modified by dermatologic response.

Subgroup analyses revealed important nuances. Obese patients and those with cardiovascular comorbidities showed consistently higher baseline SII and SIRI, consistent with literature linking these conditions to chronic systemic inflammation [12,13,14,15,16]. Notably, PLR and SII decreased significantly only in non-obese patients, suggesting that obesity and metabolic inflammation may attenuate systemic biologic responsiveness. Cardiovascular patients exhibited persistently elevated SIRI values despite dermatologic improvement, in line with evidence that cardiometabolic comorbidities contribute to ongoing low-grade inflammation [26,27]. No significant differences emerged between SR and nSR, indicating that complete and rapid skin clearance does not necessarily translate into superior systemic inflammatory control, echoing previous findings of a dissociation between cutaneous and systemic inflammation in psoriasis [12,14]. Correlation analyses confirmed only weak associations between baseline PASI and systemic inflammatory markers, with PIV being the only index demonstrating a statistically significant but weak correlation, reinforcing the concept that these biomarkers predominantly reflect systemic immuno-inflammatory burden rather than skin severity [15,16]. Overall, integrating our data with the existing literature suggests that SII and SIRI represent robust, practical indices capable of capturing systemic inflammation in psoriasis. They may be particularly useful in identifying high-risk patients, such as those with obesity or cardiovascular disease, and in monitoring long-term systemic inflammation under IL-23 inhibition. This study has several limitations: First, its retrospective and partly cross-sectional design, with the number of patients decreasing from 208 at baseline to 94 at week 156, may introduce selection bias. Importantly, this reduction reflects the nature of real-life clinical practice rather than an active selection by investigators: because data were derived from routine care, not all patients had complete laboratory assessments at every timepoint, and only those with available results could be included in each annual analysis. Second, the absence of classical inflammatory biomarkers (e.g., CRP, IL-6, TNF-α) limits mechanistic interpretation. Third, subgroup stratifications were presented (obesity, cardiovascular disease), but no multivariable analyses were conducted; therefore, observed differences may reflect confounding rather than a direct treatment effect. Fourth, no correction for multiple comparisons was applied, and some results—such as the overall trend in SIRI reduction (*p* = 0.078)—should be interpreted with caution. Fifth, the lack of a control group or comparator biologic restricts causal inference regarding the effects of guselkumab. Finally, hematologic indices, although accessible and informative, may be influenced by confounders such as obesity, cardiometabolic comorbidities, infections, or concomitant treatments, potentially affecting longitudinal trends. Despite these limitations, the study has several key strengths. It is based on a large real-world cohort, with comprehensive assessment of multiple systemic inflammatory indices. The follow-up period of up to 156 weeks represents one of the longest evaluations of guselkumab in real-life settings, providing valuable longitudinal insights. Finally, although the study population is from a single center, the relatively large sample size, the fact that all patients received the same therapy, and the heterogeneity within the cohort support the external validity of the findings.

Furthermore, the study highlights SIRI as a consistently responsive marker over long-term therapy, offering practical guidance for systemic inflammation monitoring in psoriasis patients. Prospective controlled studies are needed to clarify whether changes in systemic indices translate into measurable reductions in cardiovascular risk and whether their integration into routine clinical practice may support more personalized management strategies. Collectively, our findings demonstrate that guselkumab contributes to long-term modulation of systemic inflammation—most consistently reflected by SIRI—while highlighting the complexity and variability of biomarker trajectories in patients with psoriasis. Importantly, these indices offer a low-cost, routinely available alternative to classical biomarkers, allowing practical monitoring of systemic inflammation in real-world settings. Given that SIRI and related indices are also linked to cardiometabolic comorbidities, this study provides an important contribution to the literature, suggesting that biologic therapy may not only improve skin outcomes but also exert broader systemic benefits, potentially influencing cardiovascular risk in psoriatic patients.

## Figures and Tables

**Table 1:** Longitudinal changes in systemic inflammatory indices during guselkumab treatment.

	T0 (*n* = 208)	Week 52 (*n* = 208)	Week 104 (*n* = 129)	Week 156 (*n* = 94)	*p*-Value
**NLR**	1.92 (1.52–2.64)	1.89 (1.47–2.46)	1.97 (1.47–2.60)	1.89 (1.39–2.67)	0.180, 0.364, 0.860 * 0.335 **
**PLR**	115 (91.8–147)	113 (89.8–148)	114 (87.7–149)	108 (90.8–145)	0.475, **0.036**, 0.130 * 0.018 **
**MLR**	0.24 (0.19–0.29)	0.22 (0.18–0.28)	0.21 (0.17–0.28)	0.22 (0.17–0.26)	0.039, 0.215, **0.009** * 0.21 **
**PIV**	233 (149–369)	201 (143–376)	217 (131–357)	202 (149–326)	**0.032**, 0.580, 0.245 * 0.531 **
**SII**	452 (336–694)	445 (335–638)	488 (333–658)	458 (303–699)	0.274, 0.274, 0.56 * 0.055 **
**SIRI**	0.97 (0.67–1.43)	0.86 (0.64–1.42)	0.88 (0.62–1.28)	0.84 (0.6–1.2)	**0.015**, 0.344, 0.038 * 0.078 **

* Wilcoxon matched pairs signed rank test values refer to comparisons between baseline (T0) and weeks 52, 104, and 156. ** Friedman ANOVA test values reflect overall longitudinal trends.

**Table 2:** Longitudinal changes in inflammatory indices stratified by obesity status versus normal-weight patients.

		T0	Week 52	Week 104	Week 156	*p*-Value
**NLR**	Obese Normal weight	1.94 (1.52–2.86) 1.92 (1.52–2.58)	1.95 (1.40–2.53) 1.88 (1.51–2.42)	2.08 (1.48–2.46) 1.96 (1.48–2.64)	2.03 (1.58–2.55) 1.77 (1.38–2.70)	0.141, 0.341, 0.417 * 0.349 ** 0.525, 0.068, 0.722 * 0.196 **
**PLR**	Obese Normal weight	109 (92.6–149) 122 (91.3–144)	104 (88.7–147) 119 (90.5–149)	108 (76.7–135) 119 (94.2–154)	101 (89.4–146) 116 (91.6–146)	0.701, 0.599, 0.278 * 0.129 ** 0.224, **0.006**, **0.004** * 0.039 **
**MLR**	Obese Normal weight	0.24 (0.19–0.33) 0.24 (0.19–0.28)	0.23 (0.21–0.32) 0.21 (0.16–0.26)	0.21 (0.16–0.29) 0.21 (0.17–0.26)	0.22 (0.16–0.28) 0.21 (0.17–0.26)	0.791, 0.103, 0.072 * 0.423 ** 0.019, 0.796, 0.028 * 0.279 **
**PIV**	Obese Normal weight	238 (162–411) 227 (147–339)	250 (153–391) 189 (140–337)	236 (133–345) 210 (132–368)	256 (168–355) 189 (148–277)	0.474, 0.078, 0.488 * 0.992 ** 0.028, 0.531, 0.318 * 0.267 **
**SII**	Obese Normal weight	470 (324–699) 451 (337–664)	478 (306–649) 441 (339–621)	473 (310–636) 497 (351–705)	467 (346–701) 430 (292–682)	0.215, 0.291, 0.673 * 0.352 ** 0.622, **0.013**, 0.235 * 0.013 **
**SIRI**	Obese Normal weight	0.98 (0.72–1.66) 0.95 (0.65–1.37)	1.03 (0.72–1.61) 0.81 (0.61–1.21)	0.96 (0.63–1.27) 0.87 (0.62–1.24)	1.02 (0.62–1.54) 0.78 (0.60–1.03)	0.278, 0.056, 0.249 * 0.764 ** 0.278, 0.056, 0.249 * 0.763 **

* Wilcoxon matched pairs signed rank test values refer to comparisons between baseline (T0) and weeks 52, 104, and 156. ** Friedman ANOVA test values reflect overall longitudinal trends.

**Table 3:** Longitudinal changes in inflammatory indices according to cardiopathy status (patients with cardiopathy vs. those without).

		T0	Week 52	Week 104	Week 156	*p*-Value
**NLR**	Cardiopathy No Cardiopathy	2.57 (1.59–4.07) 1.91 (1.51–2.58)	1.96 (1.63–3.73) 1.91 (1.48–2.45)	1.83 (1.42–2.43) 1.97 (1.48–2.62)	1.95 (1.27–2.55) 1.89 (1.41–2.71)	0.373, 0.301, 0.844 * 0.896 ** 0.294, 0.194, 0.936 * 0.272 **
**PLR**	Cardiopathy No Cardiopathy	128 (98.5–152) 114 (91.8–144)	101 (91.5–134) 118 (89.8–149)	98.9 (76.7–103) 116 (88.7–150)	107 (93.0–122) 111 (90.8–147)	0.597, 0.426, 0.641 * 0.572 ** 0.366, **0.014**, 0.086 * 0.020 **
**MLR**	Cardiopathy No Cardiopathy	0.28 (0.25–0.35) 0.23 (0.18–0.28)	0.26 (0.22–0.34) 0.22 (0.17–0.27)	0.21 (0.16–0.29) 0.21 (0.17–0.28)	0.23 (0.14–0.26) 0.22 (0.18–0.26)	0.669, 0.055, **0.016** * 0.050 ** 0.035, 0.439, **0.036** * 0.393 **
**PIV**	Cardiopathy No Cardiopathy	383 (227–670) 227 (141–338)	237 (193–502) 198 (142–373)	164 (124–424) 217 (135–343)	180 (82.6–250) 203 (151–334)	0.433, 0.164, 0.253 * 0.615 ** 0.049, 0.941, 0.479 * 0.416 **
**SII**	Cardiopathy No Cardiopathy	622 (415–1102) 440 (327–673)	473 (335–586) 443 (338–649)	379 (310–636) 494 (340–660)	470 (280–583) 458 (311–699)	0.211, 0.301, 0.537 * 0.941 ** 0.505, 0.058, 0.405 * 0.067 **
**SIRI**	Cardiopathy No Cardiopathy	1.81 (0.78–2.34) 0.95 (0.65–1.36)	1.12 (0.75–2.08) 0.84 (0.62–1.23)	0.87 (0.59–2.06) 0.88 (0.62–1.23)	0.78 (0.41–1.01) 0.86 (0.61–1.22)	0.404, 0.129, 0.148 * 0.457 ** 0.021, 0.635, 0.105 * 0.068 **

* Wilcoxon matched pairs signed rank test values refer to comparisons between baseline (T0) and weeks 52, 104, and 156. ** Friedman ANOVA test values reflect overall longitudinal trends.

**Table 4:** Longitudinal changes in inflammatory indices according to clinical response (Super Responders [SR] vs. non–Super Responders [nSR]).

		T0	Week 52	Week 104	Week 156	*p*-Value
**NLR**	SR nSR	1.84 (1.49–2.58) 1.97 (1.53–2.69)	1.89 (1.49–2.54) 1.88 (4853–2.42)	1.91 (1.42–2.49) 2.02 (1.52–2.62)	1.89 (1.37–2.89) 1.89 (1.43–2.53)	0.764, 0.371, 0.806 * 0.304 ** 0.065, 0.676, 0.691 * 0.877 **
**PLR**	SR nSR	104 (84.8–134) 125 (101–149)	106 (88.9–143) 125 (91–149)	108 (81.7–143) 117 (99.5–154)	103 (89.4–142) 121 (91–149)	0.255, 0.059, 0.153 * 0.058 ** 0.761, 0.306, 0.553 * 0.358 **
**MLR**	SR nSR	0.22 (0.18–0.28) 0.24 (0.19–0.29)	0.21 (0.17–0.29) 0.23 (0.19–0.27)	0.21 (0.17–0.28) 0.22 (0.18–0.25)	0.21 (0.17–0.26) 0.22 (0.18–0.26)	0.141, 0.795, 0.432 * 0.326 ** 0.175, 0.119, **0.012** * 0.101 **
**PIV**	SR nSR	223 (149–338) 240 (149–386)	206 (149–386) 189 (135–348)	242 (132–391) 201 (132–319)	206 (153–353) 196 (144–278)	0.164, 0.469, 0.663 * 0.105 ** 0.103, 0.091, 0.225 * 0.823 **
**SII**	SR nSR	434 (312–659) 468 (362–707)	458 (347–663) 432 (315–608)	484 (347–640) 497 (328–720)	463 (302–724) 458 (317–598)	0.808, 0.132, 0.407 * 0.050 ** 0.135, 0.653, 0.929 * 0.891 **
**SIRI**	SR nSR	0.93 (0.67–1.45) 1.01 (0.66–1.41)	0.86 (0.67–1.49) 0.86 (0.62–1.29)	0.96 (0.63–1.41) 0.83 (0.61–1.17)	0.91 (0.61–1.31) 0.81 (0.58–1.07)	0.094, 0.915, 0.265 * 0.178 ** 0.101, 0.226, 0.074 * 0.322 **

* Wilcoxon matched pairs signed rank test values refer to comparisons between baseline (T0) and weeks 52, 104, and 156. ** Friedman ANOVA test values reflect overall longitudinal trends.

**Table 5:** Longitudinal changes in inflammatory indices according to prior biologic therapy exposure (bio-experienced vs. bio-naïve patients).

		T0	Week 52	Week 104	Week 156	*p*-Value
**NLR**	Bio-experienced Bio-Naïve	1.92 (1.49–2.55) 1.91 (1.52–2.81)	1.95 (1.52–2.53) 1.88 (1.41–2.41)	1.97 (1.46–2.63) 1.97 (1.48–2.51)	1.67 (1.25–2.61) 2.01 (1.62–2.73)	0.327, 0.339, 0.972 * 0.518 ** 0.317, 0.718, 0.727 * 0.631 **
**PLR**	Bio-experienced Bio-Naïve	113 (90.7–145) 121 (92.8–142)	113 (89.5–149) 113 (91.1–145)	114 (91.1–147) 114 (82.8–151)	105 (90.8–142) 119 (91.4–159)	0.268, **0.028**, 0.062 * 0.101 ** 0.792, 0.518, 0.957 * 0.097 **
**MLR**	Bio-experienced Bio-Naïve	0.23 (0.18–0.27) 0.24 (0.19–0.31)	0.22 (0.18–0.27) 0.21 (0.17–0.29)	0.22 (0.17–0.28) 0.21 (0.18–0.26)	0.21 (0.17–0.27) 0.23 (0.19–0.26)	0.427, 0.599, **0.044** * 0.045 ** 0.016, **0.004**, 0.078 * 0.036 **
**PIV**	Bio-experienced Bio-Naïve	226 (136–330) 248 (160–421)	201 (149–370) 200 (140–384)	207 (134–346) 245 (132–370)	188 (146–272) 255 (165–386)	0.244, 0.873, 0.326 * 0.855 ** 0.046, 0.261, 0.462 * 0.346 **
**SII**	Bio-experienced Bio-Naïve	449 (329–658) 452 (348–767)	454 (338–663) 431 (323–631)	494 (337–640) 476 (320–707)	247 (307–672) 559 (304–701)	0.554, 0.169, 0.318 * 0.322 ** 0.329, 0.473, 0.822 * 0.218 **
**SIRI**	Bio-experienced Bio-Naïve	0.97 (0.65–1.32) 0.97 (0.72–1.69)	0.86 (0.65–1.41) 0.89 (0.64–1.42)	0.86 (0.61–1.21) 0.95 (0.63–1.28)	0.77 (0.58–1.03) 1.02 (0.72–1.46)	0.180, 0.944, 0.098 * 0.143 ** 0.019, 0.088, 0.197 * 0.072 **

* Wilcoxon matched pairs signed rank test values refer to comparisons between baseline (T0) and weeks 52, 104, and 156. ** Friedman ANOVA test values reflect overall longitudinal trends.

**Table 6:** Correlation between PASI at baseline and systemic inflammatory markers at baseline.

Inflammatory Marker	Spearman’s ρ (Rho)	*p*-Value
NLR (W0)	0.058	0.358
PLR (W0)	0.082	0.195
MLR (W0)	0.106	0.092
PIV (W0)	0.119	**0.049**
SII (W0)	0.075	0.233
SIRI (W0)	0.121	0.100

## Data Availability

The data that support the findings of this study are available upon request from the corresponding author, M.G.

## Supplementary Materials

The following supporting information can be downloaded at https://www.mdpi.com/article/10.3390/jcm15020439/s1. Table S1: The formula of systemic inflammatory markers.

## Abbreviations

The following abbreviations are used in this manuscript:

TNF	Tumor necrosis factor
IL	Interleukin
NLR	Neutrophil-to-lymphocyte ratio
PLR	Platelet-to-lymphocyte ratio
SII	Systemic immune-inflammation index
SIRI	Systemic inflammation response index
MLR	Monocyte-to-Lymphocyte Ratio
PIV	Pan-Immune-Inflammation Value
BMI	Body mass index
PASI	Psoriasis area and severity index

## References

[1] Tashiro T., Sawada Y. (2022). Psoriasis and Systemic Inflammatory Disorders. Int. J. Mol. Sci..

[2] Chen H.H., Abed S.R. (2023). Update Aetiopathogenesis and Treatment of Psoriasis: A Literature Review. J. Dermatol. Res..

[3] Sugumaran D., Yong A.C.H., Stanslas J. (2024). Advances in psoriasis research: From pathogenesis to therapeutics. Life Sci..

[4] Blauvelt A., Chiricozzi A. (2018). The Immunologic Role of IL-17 in Psoriasis and Psoriatic Arthritis Pathogenesis. Clin. Rev. Allergy Immunol..

[5] Korman N.J. (2020). Management of psoriasis as a systemic disease: What is the evidence?. Br. J. Dermatol..

[6] Lynch M., Ahern T., Sweeney C.M., Malara A., Tobin A.M., O’Shea D., Kirby B. (2017). Adipokines, psoriasis, systemic inflammation, and endothelial dysfunction. Int. J. Dermatol..

[7] Mrowietz U., Lauffer F., Sondermann W., Gerdes S., Sewerin P. (2024). Psoriasis as a Systemic Disease. Dtsch. Arztebl. Int..

[8] Davidovici B.B., Sattar N., Prinz J., Puig L., Emery P., Barker J.N., van de Kerkhof P., Ståhle M., Nestle F.O., Girolomoni G. (2010). Psoriasis and systemic inflammatory diseases: Potential mechanistic links between skin disease and comorbid conditions. J. Investig. Dermatol..

[9] Honma M., Nozaki H. (2021). Molecular pathogenesis of psoriasis and biomarkers reflecting disease activity. J. Clin. Med..

[10] Ma R., Cui L., Cai J., Yang N., Wang Y., Chen Q., Chen W., Peng C., Qin H., Ding Y. (2024). Association between systemic immune inflammation index, systemic inflammation response index and adult psoriasis: Evidence from NHANES. Front. Immunol..

[11] Sugimoto E., Matsuda H., Shibata S., Mizuno Y., Koyama A., Li L., Taira H., Ito Y., Awaji K., Yamashita T. (2023). Impact of Pretreatment Systemic Inflammatory Markers on Treatment Persistence with Biologics and Conventional Systemic Therapy: A Retrospective Study of Patients with Psoriasis Vulgaris and Psoriatic Arthritis. J. Clin. Med..

[12] Yang Y.L., Wu C.H., Hsu P.F., Chen S.C., Huang S.S., Chan W.L., Lin S.J., Chou C.Y., Chen J.W., Pan J.P. (2020). Systemic immune-inflammation index (SII) predicted clinical outcome in patients with coronary artery disease. Eur. J. Clin. Investig..

[13] Liu Y.C., Chuang S.H., Chen Y.P., Shih Y.H. (2024). Associations of novel complete blood count-derived inflammatory markers with psoriasis: A systematic review and meta-analysis. Arch. Dermatol. Res..

[14] Zhao X., Li J., Li X. (2024). Association between systemic immune-inflammation index and psoriasis: A population-based study. Front. Immunol..

[15] Solak B., Kara R.Ö. (2024). Assessing systemic inflammatory markers in psoriasis: A retrospective study. Trop. Med. Int. Health.

[16] Murray G., Kearney N., Smith C., Carty K., Safta Y., Conlon O., Kirby B., Alsharqi A. (2025). The Systemic Immune-Inflammation Index and Its Association with Biologic Therapy Switching and Response in Psoriasis. JEADV Clin. Pract..

[17] Tamer F., Edek Y.C., Aksakal A.B. (2024). Effect of Treatment With Biologic Agents on the Novel Inflammatory Biomarkers Systemic Immune Inflammation Index and Systemic Inflammation Response Index for Psoriasis. Dermatol. Pract. Concept..

[18] Demirel Öğüt N., Ayanoğlu M.A., Koç Yıldırım S., Erbağcı E., Ünal S., Gökyayla E. (2025). Are IL-17 inhibitors superior to IL-23 inhibitors in reducing systemic inflammation in moderate-to-severe plaque psoriasis? A retrospective cohort study. Arch. Dermatol. Res..

[19] Kearney N., Gorecki P., Acciarri L., Buyze J., Akawung A., Merola J.F., Kirby B. (2025). Treatment of Plaque Psoriasis with Guselkumab Reduces Systemic Inflammatory Burden as Measured by Neutrophil/Lymphocyte Ratio, Platelet/Lymphocyte Ratio, and Monocyte/Lymphocyte Ratio: A post hoc Analysis of Three Randomised Clinical Trials. Dermatology.

[20] Zahorec R. (2021). Neutrophil-to-lymphocyte ratio, past, present and future perspectives. Bratisl. Lek. Listy.

[21] Ye J.H., Zhang Y., Naidoo K., Ye S. (2024). Neutrophil-to-lymphocyte ratio and platelet-to-lymphocyte ratio in psoriasis: A systematic review and meta-analysis. Arch. Dermatol. Res..

[22] Dincer Rota D., Tanacan E. (2021). The utility of systemic-immune inflammation index for predicting the disease activation in patients with psoriasis. Int. J. Clin. Pract..

[23] Liu Z., Zheng L. (2024). Associations between SII, SIRI, and cardiovascular disease in obese individuals: A nationwide cross-sectional analysis. Front Cardiovasc. Med..

[24] Lin Y., Sun J., Fang S., Li C., Yang X., Yuan H., Zhang Z. (2025). Association between novel inflammatory biomarkers SII, SIRI, and obesity in sedentary adults: NHANES 2007–2020. Sci. Rep..

[25] Di Cesare A., Rosi E., Trovato E., Pescitelli L., Panduri S., Margiotta F.M., Michelucci A., Capalbo E., Dragotto M., Magnano M. (2025). Therapeutic Modulation of Peripheral Blood Cells And Inflammatory Indices During 52 Weeks Of Risankizumab In Responder Patients with Moderate-to-Severe Psoriasis: Results From A Multicenter Prospective Study. Dermatol. Pract. Concept..

[26] Dziedzic E.A., Gąsior J.S., Tuzimek A., Paleczny J., Junka A., Dąbrowski M., Jankowski P. (2022). Investigation of the Associations of Novel Inflammatory Biomarkers—Systemic Inflammatory Index (SII) and Systemic Inflammatory Response Index (SIRI)—With the Severity of Coronary Artery Disease and Acute Coronary Syndrome Occurrence. Int. J. Mol. Sci..

[27] Wang C., Yan W., Ren M., Zhong L. (2024). Screening significance of systemic immune-inflammation index (SII) and systemic inflammation response index (SIRI) in coronary heart disease of symptomatic youth. Immun. Inflamm. Dis..

