# Supplementary material for: Long-Term Impact of Guselkumab on Systemic Inflammation Indices in Moderate-to-Severe Psoriasis
Source: J Clin Med. 2026 Jan 6;15(2):439. doi: 10.3390/jcm15020439 (PMC12842056; doi:10.3390/jcm15020439)
Supplement: Supplementary file 1 [file jcm-15-00439-s001.zip › jcm-4063674-supplementary.pdf]

Supplementary Table S1. The formula of systemic inflammatory markers

**Inflammatory Marker**	**The formula**
NLR	Neutrophil count/lymphocyte count
PLR	Platelet count/lymphocyte count
MLR	Monocyte count/lymphocyte cout
PIV	Neutrophil count × monocyte count × platelet/lymphocyte count
SII	Neutrophil count × platelet count/ lymphocyte count
SIRI	Neutrophil count × monocyte count/lymphocyte count
